# Restricted Morphological and Behavioral Abnormalities following Ablation of β-Actin in the Brain

**DOI:** 10.1371/journal.pone.0032970

**Published:** 2012-03-05

**Authors:** Thomas R. Cheever, Bin Li, James M. Ervasti

**Affiliations:** Department of Biochemistry, Molecular Biology and Biophysics, University of Minnesota, Minneapolis, Minnesota, United States of America; Medical College of Georgia, United States of America

## Abstract

The local translation of β-actin is one mechanism proposed to regulate spatially-restricted actin polymerization crucial for nearly all aspects of neuronal development and function. However, the physiological significance of localized β-actin translation in neurons has not yet been demonstrated *in vivo*. To investigate the role of β-actin in the mammalian central nervous system (CNS), we characterized brain structure and function in a CNS-specific β-actin knock-out mouse (CNS-*Actb*KO). β-actin was rapidly ablated in the embryonic mouse brain, but total actin levels were maintained through upregulation of other actin isoforms during development. CNS-*Actb*KO mice exhibited partial perinatal lethality while survivors presented with surprisingly restricted histological abnormalities localized to the hippocampus and cerebellum. These tissue morphology defects correlated with profound hyperactivity as well as cognitive and maternal behavior impairments. Finally, we also identified localized defects in axonal crossing of the corpus callosum in CNS-*Actb*KO mice. These restricted defects occurred despite the fact that primary neurons lacking β-actin in culture were morphologically normal. Altogether, we identified novel roles for β-actin in promoting complex CNS tissue architecture while also demonstrating that distinct functions for the ubiquitously expressed β-actin are surprisingly restricted *in vivo*.

## Introduction

The localized polymerization of actin has critical functions in neurons where it contributes to almost every stage of neuronal development from migration [1] and growth cone guidance [2] to dendritic spine remodeling and learning [3]. Although the mechanisms regulating localized actin polymerization in neurons remain incompletely understood, current data support two non-mutually exclusive models. The first model relies on the regulated activity of a plethora of actin binding proteins, while the second model focuses on local synthesis of β-actin to mediate localized actin polymerization in neurons. β-actin is one of six actin isoforms expressed in mammalian cells and is the only isoform thought to be locally translated [4]–[6].

The molecular mechanism of the latter model has been extensively characterized in cell culture and is dependent on a 54 nucleotide sequence in the 3′ untranslated region of β-actin mRNA called the “zipcode” [7]. The zipcode sequence of β-actin mRNA is bound by zipcode binding protein-1 (ZBP1) which mediates the transport of β-actin mRNA from the cell body to the growth cone while also inhibiting its translation [8]–[10]. When attractive guidance cues are received at one side of the growth cone, a signaling cascade is initiated leading to the release and local translation of β-actin [9], [11]–[13]. However, growth cones are believed to constitutively express high concentrations of monomeric actin, and a general role for local translation in early growth cone guidance is controversial [14], [15]. Furthermore, the significance of this mechanism *in vivo* has yet to be established. A knock-out of the mouse ZBP1ortholog *Imp1* exhibited high perinatal lethality but no reported brain pathology [16], while motor neuron specific β-actin knock-out mice presented with no defects in motor neuron function or axonal regeneration [17].

In order to investigate the role of β-actin in the development and function of the mammalian CNS, we generated a central nervous system specific β-actin knock-out mouse (CNS-*Actb*KO). β-actin was rapidly and efficiently ablated from the brains of CNS-*Actb*KO mice while γ- and α_sm_-actin isoforms were upregulated to maintain total actin levels during development. However, nearly two thirds of these mice died postnatally. Examination of brains from viable CNS-*Actb*KO mice revealed specific defects in the morphology of the cerebellum and hippocampus that correlated with hyperactivity, poor cognitive performance in the Morris water maze, and maternal behavior defects. CNS-*Actb*KO mice also exhibited partial agenesis of the corpus callosum. Cultured primary neurons from CNS-*Actb*KO embryos exhibited only limited morphological defects however. Together, our study reveals a previously unreported role for β-actin in the morphogenesis of the hippocampus, corpus callosum, and cerebellum, while demonstrating that the local translation of β-actin may have a more restricted role in nervous system development and function than previously thought.

## Results

### Actin isoform expression in CNS-*Actb*KO brains

**Figure 1:**
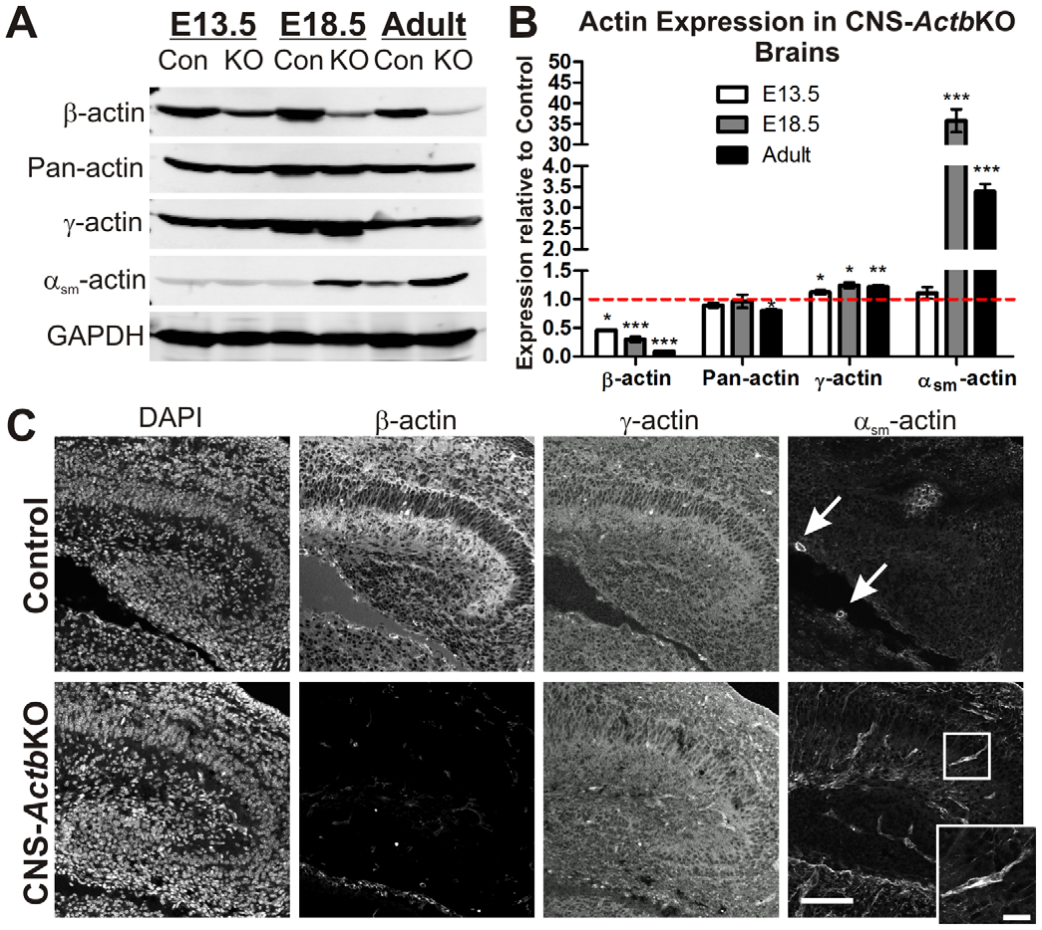
Conditional ablation of β-actin in the mouse central nervous system. (**A–B**) Quantitative Western blot analysis of actin isoform expression in control and CNS-*Actb*KO brains at embryonic day (E)13.5, 18.5 and in adults (6–9 months). Data plotted as mean ± standard error of the mean with an *n*≥3 per genotype per age point. (**C**) Immunofluorescence with actin isoform-specific antibodies on saggital sections of the hippocampus at E18.5. β-actin staining was dramatically reduced in CNS-*Actb*KO brain sections. γ-actin staining gave a similar localization pattern to β-actin on co-labeled control sections and was still present in CNS-*Actb*KO embryos. α_sm_-actin was restricted to blood vessels in control brains (arrows) but was prominent in select cells in CNS-*Actb*KO brain sections (see inset, inset scale bar 25 µm). Scale bar 100 µm. * indicates *p*<0.05, ** indicates *p*<0.01, *** indicates *p*<0.001.

In order to examine the role of β-actin in the CNS, floxed *Actb* mice [18] were crossed to the transgenic Nestin-Cre line [19], [20] to generate CNS-specific β-actin knock-out mice (CNS-*Actb*KO). Brains from embryonic day (E) 13.5, E18.5, and adult control and CNS-*Actb*KO mice were analyzed via quantitative Western blot analysis to confirm β-actin ablation (Figure 1A–B). At E13.5, approximately three days after Cre expression and DNA recombination, β-actin levels in CNS-*Actb*KO brains were decreased 53.8% and further reduced to 30% of controls at E18.5. Adult CNS-*Actb*KO mice exhibited a 90.9% reduction of β-actin relative to controls, with the remaining β-actin likely due to the presence of non-CNS derived cells (Figure 1C). Using a pan-actin antibody that recognizes all actin isoforms, we measured similar levels of total actin in CNS-*Actb*KO and control embryos throughout development although we did observe a small but statistically significant decrease in total actin expression in adult brains (Figure 1A–B). The most closely related actin isoform to β-actin, γ-actin, was upregulated on average 19.5% at all time points analyzed. Surprisingly, the largest increase in actin isoform expression was measured for vascular smooth muscle α-actin (α_sm_-actin), which was upregulated 35-fold at E18.5 and 3.4-fold in adults.

To examine the localization of actin in CNS-*Actb*KO brains, we performed immunofluorescence microscopy with actin isoform-specific antibodies on brain sections from E18.5 control and CNS-*Actb*KO embryos. While β-actin was expressed throughout the hippocampal region in control embryos, signal from CNS-*Actb*KO sections in the hippocampus was absent (Figure 1C). The pattern of γ-actin staining in CNS-*Actb*KO sections was indistinguishable from β- and γ-actin staining in control sections (Figure 1C). α_sm_-actin was only detectable around vasculature in controls (Figure 1C arrows), but was surprisingly found in select populations of cells throughout the hippocampus in CNS-*Actb*KO embryos (Figure 1C bottom right panel and inset).

### Perinatal lethality and gross morphological phenotypes in CNS-*Actb*KO mice

**Figure 2:**
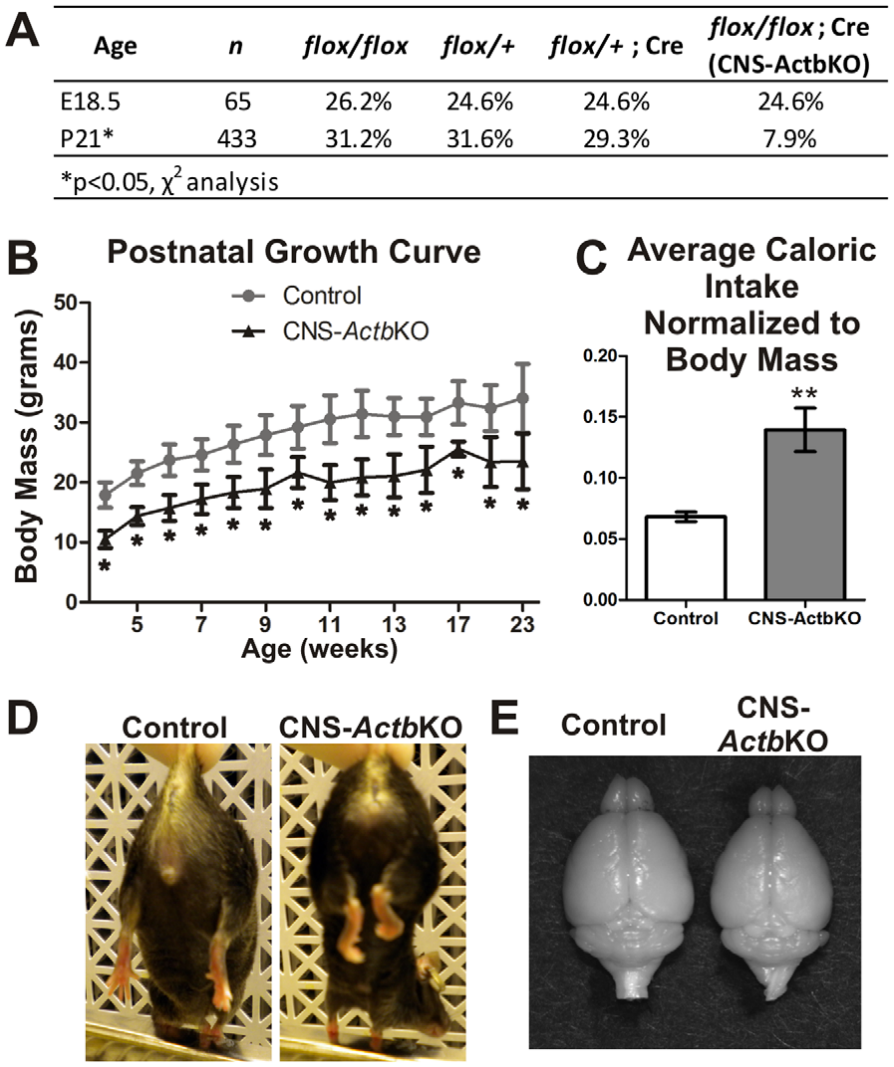
Gross characterization of CNS-*Actb*KO mice. (**A**) While the expected number of CNS-*Actb*KO embryos were present at E18.5, nearly 2/3's died between birth and weaning at P21. (**B**) Surviving CNS-*Actb*KO mice were significantly smaller than control littermates from weaning at P21 through adulthood, although they followed a similar trend in growth. (**C**) Despite their reduced mass, CNS-*Actb*KO mice had a significantly higher caloric intake than control mice when normalized to body weight. (**D**) Hind-limb clasping/contractures were present in CNS-*Actb*KO mice indicating neurological dysfunction. (**E**) Brains from CNS-*Actb*KO mice appeared grossly normal and were proportionate in size to the smaller body mass of CNS-*Actb*KO mice. *n*≥3 mice for each genotype. Data plotted as mean ± standard error of the mean. * indicates *p*<0.05, **indicates *p*<0.01.

We next determined whether the increased expression of γ- and α_sm_-actin was able to compensate for the ablation of β-actin and maintain the viability of CNS-*Actb*KO mice. The expected number of CNS-*Actb*KO embryos were found immediately prior to birth at E18.5, but nearly two thirds were lost before weaning at postnatal day 21 (P21) due to unknown causes (Figure 2A). E18.5 CNS-*Actb*KO embryos were morphologically indistinguishable from controls and of a similar mass (control: 1.094±0.040 g, CNS-*Actb*KO: 1.188±0.026 g). The small number of CNS-*Actb*KO mice that escaped perinatal lethality were on average 32.4% smaller than control littermates and maintained this decreased mass throughout adulthood despite having a caloric intake two times greater than controls (Figure 2B–C). Surviving CNS-*Actb*KO mice also exhibited hind-limb clasping or contractures (Figure 2D) which are generally indicative of neuromuscular dysfunction. Adult CNS-*Actb*KO mice were otherwise morphologically unremarkable however, and lived normal lifespans. Isolated brains of CNS-*Actb*KO mice revealed no obvious abnormalities other than a slightly decreased size which was proportional with their reduced body size (Figure 2E).

### Restricted histopathology in CNS-*Actb*KO brains

**Figure 3:**
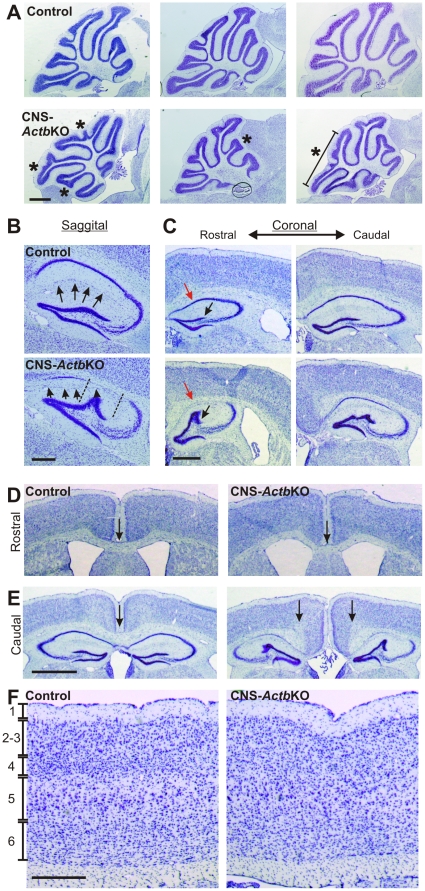
Histological abnormalities in CNS-*Actb*KO brains. (**A**) Midsaggital sections through the cerebellum revealed variable abnormalities in the foliation pattern of the cerebellum. Aberrantly positioned folia are marked by asterisks. Scale bar 1 mm. (**B**) Morphological abnormalities in the hippocampus as seen in saggital sections. Arrows indicate the position of the hippocampal fissure normally found between the dentate gyrus and CA1 neuronal layer. In CNS-*Actb*KO mice, the hippocampal fissure was displaced (arrows) which correlated with an abnormal invagination of the dentate gyrus. The region corresponding to this displacement is indicated by dashed lines which also reveals a dramatic decrease in cell body staining in the CA1 neuronal layer. Scale bar 0.5 mm. (**C**) The abnormal morphology of the hippocampus in CNS-*Actb*KO mice is further illustrated in coronal sections where the dentate gyrus displacement (black arrows) and decrease in CA1 region staining (red arrows) can also be seen. Scale bar 0.5 mm. (**D**) Axons of the corpus callosum crossed the midline normally in rostral sections from CNS-*Actb*KO brains (arrows) but failed to cross the midline in more caudal sections (arrows in **E**). Scale bars in D–E, 0.5 mm. (**F**) The lamination of cortical layers 1–6 was preserved in 6 month old control and CNS-*Actb*KO mice. Scale bar 0.5 mm.

Although surviving CNS-*Actb*KO mice presented with only limited gross phenotypes, examination of CNS-*Actb*KO brains histologically revealed several morphological abnormalities in select areas of the brain. Midsaggital sections through the adult cerebellum and stained with Cresyl violet revealed abnormal foliation patterns (denoted by asterisks in Figure 3A) that varied from extensive (Figure 3A-left panel), to the aberrant positioning of only a single folia (Figure 3A-middle panel). Interestingly, lamination of the cerebellum appeared unperturbed in the absence of β-actin, with no gross derangement of the molecular or granule cell layer.

Examination of the hippocampus in adult CNS-*Actb*KO mice also revealed prominent abnormalities. In saggital sections of the hippocampus, the dorsal arm of the dentate gyrus was abnormally positioned and approached the CA1 pyramidal neuron layer (Figure 3B). In addition, the hippocampal fissure (denoted by arrows in Figure 3B) intersected with a portion of the CA1 pyramidal neuron layer correlating spatially with the invasion of the dentate gyrus (delineated by dashed lines in Figure 3B). A dramatic decrease in cell body staining in the CA1 pyramidal neuron layer was also apparent in the region invaded by the dentate gyrus and hippocampal fissure (area between dashed lines). These abnormalities were also apparent in coronal sections where the derangement of the dorsal arm of the dentate gyrus (black arrow in Figure 3C) and decreased staining in the CA1 neuronal layer (red arrow in Figure 3C) can be seen.

CNS-*Actb*KO mice also presented with partial agenesis of the corpus callosum. While the callosal axons from control and CNS-*Actb*KO mice completely crossed the midline in rostral sections (Figure 3D), axons from CNS-*Actb*KO mice stopped short in more caudal sections (arrows in Figure 3E, right panel) and appeared to assemble into probst bundles indicative of growth cone aggregation due to an inability to traverse a substrate. Interestingly, the agenesis of the corpus callosum appeared to occur at levels that overlay the hippocampus, suggesting a possible interaction between the aberrant morphology of the hippocampus and corpus callosum. No other features of the brain appeared disturbed, including the cortex which was laminated properly into the six cortical layers (Figure 3F). Thus, abnormal tissue architecture in CNS-*Actb*KO brains was apparent only in a subset of brain structures.

### Behavioral abnormalities in CNS-*Actb*KO mice

**Figure 4:**
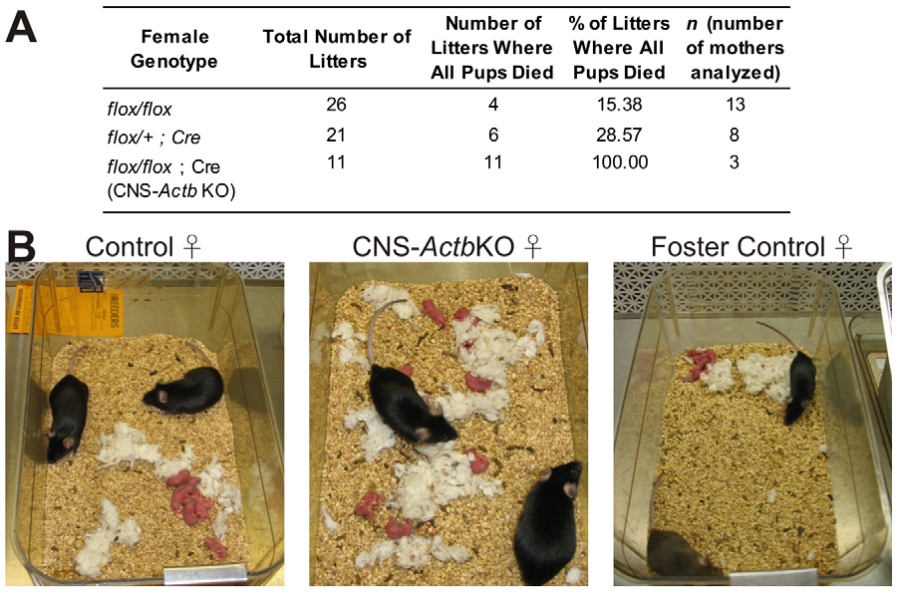
Maternal behavior deficit in CNS-*Actb*KO mice. (**A**) Pups born to CNS-*Actb*KO females did not survive longer than one day. (**B**) CNS-*Actb*KO mothers exhibited a pup retrieval deficit. While control mothers retrieved pups into a nest, pups born to CNS-*Actb*KO females were scattered throughout the cage. The pup retrieval deficit was independent of the pups however, as pups born to CNS-*Actb*KO females were retrieved normally by foster control females.

CNS-*Actb*KO mice exhibited no overtly abnormal cage behaviors with one striking exception. No pups born to a CNS-*Actb*KO female survived longer than one day regardless of genotype (Figure 4A). This maternal behavior defect correlated with an apparent pup retrieval deficit (Figure 4B) which may explain the pup mortality. Importantly, the maternal behavior defect observed was not due to the pups themselves, as pups born to CNS-*Actb*KO mothers could be fostered to control females where they were retrieved and survived (Figure 4B, right).

**Figure 5:**
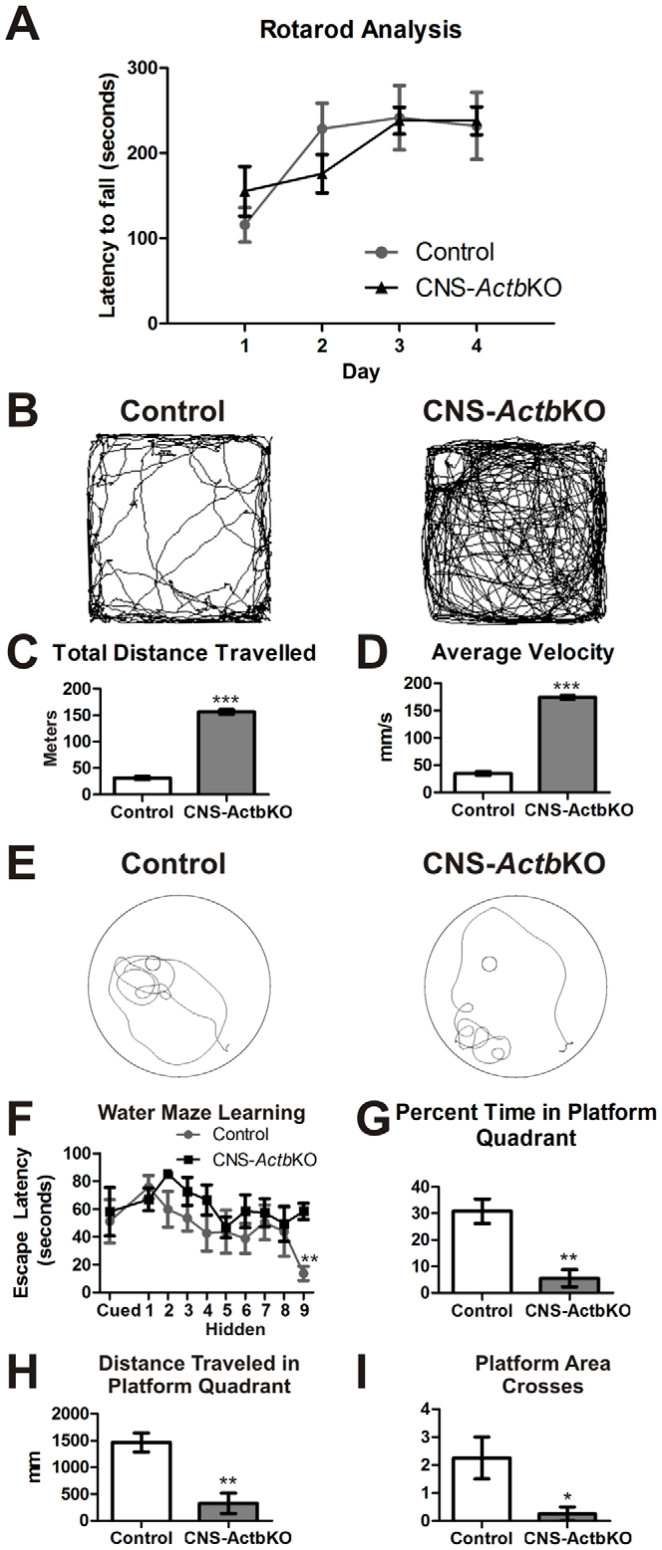
CNS-*Actb*KO mice exhibited hyperactivity and decreased performance in the Morris water maze. (**A**) CNS-*Actb*KO mice performed comparably to controls in a four day Rotarod assay experiment. Control and CNS-*Actb*KO animals were age matched and between 4–6 months old. Data plotted as mean ± standard error of the mean. (**B**) Five minute traces from control and CNS-*Actb*KO mice in an Open Field Activity Assay. (**C–D**) CNS-*Actb*KO mice traveled significantly greater distances and at significantly greater velocities than control mice. (**E**) Representative 30 second traces from control and CNS-*Actb*KO mice during the probe trial of a Morris water maze test. (**F**) CNS-*Actb*KO mice performed similarly to control mice with a cued platform and followed a similar learning curve until the final day of the hidden platform learning phase, at which time controls found the platform significantly faster. (**G–H**) In the probe trial, CNS-*Actb*KO mice spent a significantly smaller percentage of time in the platform quadrant and traveled significantly less distance in that quadrant. (**I**) CNS-*Actb*KO mice also crossed an area 50% larger than that of the actual platform significantly less times than control mice. *n* = 4 male mice for each genotype. * indicates *p*<0.05, ** *p*<0.01, *** *p*<0.001.

In order to determine if the observed cerebellar histological anomalies resulted in behavioral phenotypes, cerebellar function was assessed using the Rotarod apparatus. CNS-*Actb*KO mice performed similarly to controls however, suggesting cerebellar functions in motor performance are largely intact (Figure 5A). We also conducted an Open Field activity assay to examine motor function and basal activity levels of CNS-*Actb*KO mice. Surprisingly, CNS-*Actb*KO mice exhibited a profound hyperactive phenotype. Representative traces of mouse activity from the first five minutes of a fifteen minute trial are shown in Figure 5B. CNS-*Actb*KO mice traveled significantly farther and at a significantly greater average velocity than control mice (Figure 5C–D). The profound hyperactivity may explain the significant increase in caloric demand observed in CNS-*Actb*KO mice (Figure 2C) and the reduced size of these animals throughout their lifetime (Figure 2B). Interestingly, the hippocampus has also been implicated in the regulation of mouse basal activity, suggesting that the morphological abnormalities in the hippocampus could contribute to the hyperactive phenotype observed [21], [22].

To more directly examine hippocampal and cognitive function, CNS-*Actb*KO and control mice were subjected to a Morris water maze. CNS-*Actb*KO mice performed similarly to controls with a cued platform, suggesting that vision is not likely impaired in these animals (Figure 5F). During a nine day hidden platform training series, CNS-*Actb*KO mice appeared to perform comparably to controls until the last day, at which time CNS-*Actb*KO mice took significantly longer than controls to find the platform (Figure 5F). Traces from the probe trial revealed that CNS-*Actb*KO mice employed an unusual search pattern (Figure 5E) and spent a significantly smaller percentage of time in the platform quadrant compared to controls (Figure 5G). This was also corroborated by quantification of distance traveled in the platform quadrant (Figure 5H) and platform area crosses (Figure 5I), both of which were significantly decreased in CNS-*Actb*KO mice. The abnormal performance of CNS-*Actb*KO mice suggests these mice suffer from cognitive dysfunction with a possible basis in aberrant hippocampal organization.

### CNS-*Actb*KO hippocampal neurons are comparable to controls in culture

**Figure 6:**
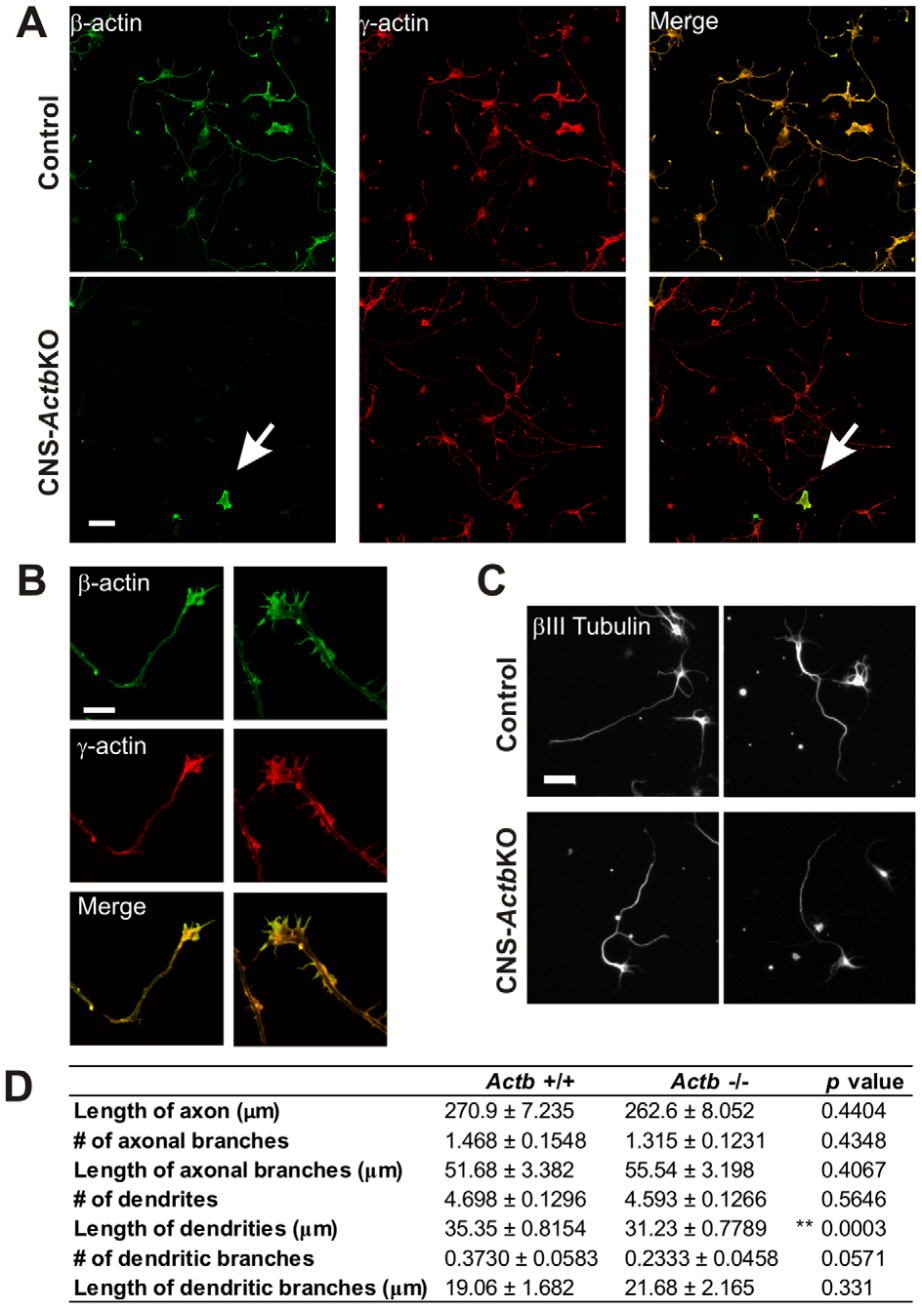
CNS-*Actb*KO primary neurons exhibit normal morphology. (**A**) Hippocampal neurons derived from E15.5 control and CNS-*Actb*KO embryos and cultured for 3 days *in vitro*. Neurons were stained with antibodies specific for β- and γ-actin. β-actin was efficiently ablated in hippocampal neuronal cell bodies and growth cones (**B**) from CNS-*Actb*KO embryos. Arrows in the bottom panels in (**A**) indicates a non-neuronal cell expressing β-actin as a positive control. (**C**) Representative images of control and CNS-ActbKO neurons stained with a βIII-tubulin antibody for morphological characterization (**D**). Scale bars 20 µm in (**B**), 50 µm in all other panels.

While the restricted nature of histological abnormalities in CNS-*Actb*KO brains was unexpected, agenesis of the corpus callosum is often linked to axon guidance defects [23] where β-actin is believed to have an essential role. In order to investigate whether the corpus callosum agenesis was due to defective neuronal development, we cultured primary hippocampal neurons from control and CNS-*Actb*KO embryos to examine neuronal morphology. Hippocampal neurons derived from E15.5 CNS-*Actb*KO embryos and cultured three days *in vitro* were null for β-actin by immunofluorescence (Figure 6A). Immunolocalization of β- and γ-actin in control neurons revealed that both cytoplasmic actin isoforms colocalized throughout the neuronal cell body (Figure 6A) and growth cone (Figure 6B).

We next analyzed morphological parameters in hippocampal neurons to determine if the absence of β-actin adversely affected neuritogenesis or differentiation *in vitro*. Quantification of neurons labeled with a βIII-tubulin antibody revealed no significant differences in the length of axon or axon branches, number of axonal branches, number of dendrites or dendritic branches, or length of dendritic branches (Figure 6C–D). However, we did observe a small but significant decrease in the length of primary dendrites (Figure 6D). The number of dendritic branches was also reduced in CNS-*Actb*KO neurons, but did not reach statistical significance (Figure 6D). Overall, the lack of dramatic effects on neuronal differentiation *in vitro* suggests β-actin is not essential in neurons for normal morphogenesis.

## Discussion

Here we report the role of β-actin in the mammalian brain and identify distinct contributions of β-actin to normal brain structure and function. Although β-actin is believed to comprise one-half to two-thirds of the cytoplasmic actin in the mammalian brain [24], [25], we showed that total actin levels are maintained during development in CNS-*Actb*KO embryos due to an upregulation of γ- and α_sm_-actin. However, CNS-*Actb*KO mice presented with significant perinatal lethality, with only 32% surviving to adulthood. Characterization of brain histology in CNS-*Actb*KO mice revealed dramatic perturbations in the structure of the cerebellum and hippocampus that correlated with behavioral and cognitive abnormalities. Finally, CNS-*Actb*KO mice exhibited partial agenesis of the corpus callosum while cultured neurons from CNS-*Actb*KO embryos had largely normal morphology.

The most prominent phenotype we identified in CNS-*Actb*KO mice was the disrupted architecture of the hippocampus and cerebellum. Interestingly, gross neuronal lamination in the hippocampus, cerebellum, and cortex (Figure 3) was normal, indicating neuronal migration along radial tracts is not likely β-actin dependent. Instead, the morphological abnormalities observed appear to stem primarily from defects in tissue architecture, suggesting impaired regulation of localized cell-cell or cell-extracellular matrix interactions. Alternatively, the outgrowth of cerebellar folia [26] and folding of the developing hippocampus [27], [28] are reminiscent of the complex tissue remodeling that occurs during neural tube closure in embryonic development. Hingepoints at developing tissue folds form when select cells undergo apical constriction mediated by localized actin-myosin contraction [29], [30]. A longstanding question in the field has been how actin and myosin are localized to the apical domain of hingepoint cells [30], [31]. One intriguing hypothesis is that perhaps the local translation of β-actin may facilitate the localized accumulation of actin. Interestingly, Dugina *et al.* previously reported β-actin was strongly enriched at the cleavage furrow in dividing cells in culture while γ-actin was localized ubiquitously along the cortical cytoskeleton, providing precedent for such a role of β-actin in a similar cellular constriction process [32]. The fact that CNS-*Actb*KO embryos do not present with neural tube closure defects is most likely because formation of hingepoints during neurulation occurs between E8.5 and E11, while Nestin-Cre expression commences at approximately E10.5 [20], [29], [33]. Pre-existing β-actin mRNA and protein present before Cre expression are thus likely sufficient to facilitate proper neural tube development.

The actin filaments involved in mediating tissue morphogenic movements must be dynamic to facilitate cellular shape changes, but at other times more stable to endure mechanical strain encountered during these movements [34]. Although actin binding proteins are hypothesized to play the primary role in regulating the dynamics and stability of actin filaments, the composition of actin itself within filaments may also contribute. A recent study by Bergeron *et al.* characterized the biochemical properties of individual actin isoforms and found that β-actin exhibits more rapid polymerization, phosphate release, and depolymerization than γ-actin, suggesting β-actin may be the more dynamic cytoplasmic actin isoform [35]. Thus, it is plausible that cells may also spatiotemporally regulate the properties of actin filaments by controlling their relative composition of β- and γ-actin, with filaments more rich in β-actin being relatively more dynamic. It also warrants mention however that α-actin filaments are reported to be more stable than β- or γ-actin filaments [36], [37]. Given the significant upregulation of α_sm_-actin in CNS-*Actb*KO brains (Figure 1C), the incorporation of the more stable α_sm_-actin into otherwise more dynamic filaments could also perturb tissue morphogenesis and lead to the abnormal brain architecture seen in CNS-*Actb*KO mice.

Intriguingly, the altered morphology of specifically the hippocampus may also inform on the mechanism of the partial corpus callosum agenesis in CNS-*Actb*KO mice. Corpus callosum agenesis is a relatively complex phenomenon with significant contributions derived from neuronal axon guidance as well as glial support populations and axonal growth substrates [23], [38]. Pioneer callosal axons begin crossing the midline in most regions of the mouse brain at E15.5 [39]. In regions where the corpus callosum lies above the hippocampus, callosal axons are hypothesized to follow the pre-existing hippocampal commissures derived from hippocampal neuron axons to facilitate midline crossing [23], [40]. The dorsal hippocampal commissure, which lies immediately ventral to the corpus callosum in the region depicted in Figure 3E, also does not cross the midline in CNS-*Actb*KO brains, and thus the later arriving callosal axons may fail to cross because their normal growth substrate is absent. While defects in axon guidance due to the inability to locally translate β-actin may contribute to the axonal defects observed, disruptions in local glial populations or growth substrates caused by ablation of β-actin may also contribute substantially to the phenotypes observed, and perhaps explain the highly restricted nature of the defect.

The data presented here along with our previous report characterizing the role of β-actin in motor neuron function and axonal regeneration [17] further suggests that distinct roles for β-actin may be unexpectedly confined to select nervous system cell populations. By using a CNS-wide conditional knock-out approach as described here with a Nestin-Cre line, we were able to identify cell populations where β-actin appears to be particularly important. Although CNS-*Actb*KO mice exhibit significant perinatal lethality hindering studies in adult mice, future studies could take advantage of more selective Cre lines to help further define the molecular mechanisms of the phenotypes described here and the role of β-actin and other actin isoforms in cytoskeletal regulation *in vivo*.

## Materials and Methods

### Ethics Statement

The experimental protocols in this study were reviewed and approved by the University of Minnesota Institutional Animal Care and Use Committee (IACUC) and approved on August 13^th^, 2010 (IACUC Protocol #0907A69551).

### Mouse Lines, breeding, and ethics statement

CNS-*Actb*KO mice were generated by crossing a floxed *Actb* mouse line [18] to the Nestin-Cre transgenic line (Stock# 003771, Jackson Labs, Bar Harbor, ME) as described previously [17]. Homozygous floxed animals lacking the Cre transgene were used as controls. Because of the scarcity of CNS-*Actb*KO animals and a maternal behavior defect in CNS-*Actb*KO females, breeding of *Actb flox/+*; Nestin-cre animals to *Actb flox/flox* animals was required. For timed matings, the morning a vaginal plug was found was designated E0.5.

### Brain lysate preparation and quantitative Western blot analysis

Control and CNS-*Actb*KO embryo and adult (6–9 month old) mouse brains were snap frozen and pulverized in liquid nitrogen. Pulverized tissue was solubilized in a 1∶10 (m∶V) dilution of SDS lysis buffer (1% SDS, 5 mM EGTA, 1 mM Benzamadine, 10 µM Leupeptin, 0.2 mM PMSF) at 100°C for 5 minutes. Lysates were then cleared by centrifugation at 25,000×g for 2 minutes. The resulting supernatant was collected and analyzed with the Bio-Rad D_C_ protein assay kit to determine protein concentration. 25 mg of control and CNS-*Actb*KO brain lysates were then run side by side and in duplicate on 8–16% SDS-polyacrylamide gels, transferred to PVDF membrane, and blocked for 1 hour in 5% milk. The following primary antibodies were used: C4, a pan-actin antibody that recognizes all actin isoforms (LMAB-C4, Seven Hills Bioreagents, Cincinatti, OH, 1∶1000), anti- β-actin antibody AC15 (A1978, Sigma Aldrich, St. Louis, MO, 1∶1000), anti-γ-actin antibody 2–4 ([41], 1∶1000) and anti-α_sm_-actin antibody 1A4 (A5228, Sigma Aldrich, 1∶500). All membranes were also incubated with an anti-GAPDH antibody (G9545, Sigma Aldrich, 1∶2000) as a loading control. Infrared dye conjugated secondary antibodies were purchased from Li-Cor Biosciences. The infrared signals from duplicate blots were quantitatively measured with the Li-Cor Odyssey Imager system (Li-Cor Biosciences, Lincoln, NE, USA), normalized to the loading control signal and averaged.

### Brain isolation, histology and immunofluorescence

Brains for histology were isolated from age matched adult (4–18 month old) control and CNS-*Actb*KO mice and perfused, post-fixed and cryoprotected as described previously [17]. Brains were then blocked in the desired orientation and frozen in OCT (TissueTek, Torrance, CA) on dry ice. Sixteen micron cryosections were cut and stored at −20°C until processing for histology or immunofluorescence. For histology, sections were equilibrated to room temperature and stained with 0.1% Cresyl Violet acetate (C1791, Sigma Aldrich, St. Louis, MO) following standard protocols. Stained sections were mounted with Eukitt (03989, Sigma Aldrich).

For immunofluorescence with actin isoform-specific antibodies, sections were post-fixed in 100% Methanol and processed as described previously [17]. For β- and γ-actin staining, sections were co-labeled with directly conjugated anti- β-actin AC15 (ab6277; Abcam, Cambridge MA, 1∶75) and directly conjugated mouse monoclonal anti-γ-actin (Clone 1-37-Alexa568; [18], [41], 1∶50). Anti-α_sm_-actin 1A4 was used to localize α_sm_-actin in brain sections (A5228; Sigma Aldrich, 1∶400) with an anti-mouse Alexa Fluor 488 conjugated secondary antibody (Invitrogen, Carlsbad, CA, 1∶500). Coverslips were mounted with SlowFade Gold antifade reagent with DAPI (S36938; Invitrogen). Images were acquired on an Olympus FluoView FV1000 laser scanning confocal microscope at the Biomedical Image Processing Laboratory with identical settings between control and CNS-*Actb*KO sections. Images were further processed identically with Adobe Photoshop.

### Behavioral Assays

#### Rotarod

An accelerating Rotarod apparatus (Accelerating Model, Ugo Basile Biological Research Apparatus, Varese, Italy) was used to assess motor coordination in gender matched 6 month old control and CNS-*Actb*KO mice (*n* = 8 controls and 6 CNS-*Actb*KO mice). The Rotarod was configured to accelerate from 4–40 RPM in five minutes, and continue at the maximal speed for another five minutes. Mice were tested for four consecutive days with four trials per day and 10 minute rest intervals between each trial.

#### Open Field Activity Assay

Activity of age matched adult (6–9 month old) male control (*n* = 4) and CNS-*Actb*KO (*n* = 4) mice in a 50×50×40 cm test cage was videotaped and analyzed using the Topscan System (Clever System). Mice were allowed to explore the cage for 15 minutes while their activity was tracked.

#### Water Maze

A water maze analysis was conducted in a 1.2 m diameter opaque pool with water maintained at 24±0.5°C. The testing room was decorated with posters and other fixtures to serve as spatial cues. Age matched adult (6–9 month old) male control (*n* = 4) and CNS-*Actb*KO (*n* = 4) mice were trained to locate a 10 cm platform submerged 0.5 cm under the water in an arbitrary quadrant during 90 second training trials. On the first day, the platform was raised to the surface of the water and fitted with a 15 cm flag to serve as a cued control for possible visual differences between control and CNS-*Actb*KO animals. Hidden platform learning sessions were conducted on the following nine days, where mice performed two 90 second trials per day separated by 30 minutes. Mice were started from randomized positions approximately equidistant from the hidden platform. For the probe trial, the platform was removed and mice were started from a novel starting position for a 30 second probe trial. Video data was analyzed with the Topscan Video Analysis System (Clever System).

#### Food Consumption

Food consumption from adult (4–6 month old) male control and CNS-*Actb*KO mice (*n* = 4 for each genotype) was analyzed by weighing the amount of food initially placed in the cage and the amount consumed per 24 hours for three consecutive days. The data from the three days was then averaged.

### Culturing Primary Hippocampal Neurons

Primary hippocampal neurons from E15.5 embryos were isolated and cultured individually as described previously [42] with modifications listed below. Embryos were genotyped from tail snip lysates following protocols described previously [17]. 80,000 neurons were plated in 35 mm dishes containing acid washed coverslips coated with Poly-D-Lysine (100 µg/ml) and laminin (4 µg/ml). Neurons were plated in neuronal plating media (MEM with Earle's salts, 10 mM HEPES, 10 mM sodium pyruvate, 0.5 mM glutamine, 12.5 µM glutamate, 10% FBS, and 0.6% glucose). Four to six hours after plating, the media was replaced with neuronal growth media (Neurobasal media with B27 supplement, 0.5 mM glutamine, 12.5 mM glutamate, 1× penicillin/streptomycin (P433, Sigma Aldrich)).

For all experiments, neurons were cultured for three days *in vitro* and then fixed in 4% PFA in PBS warmed to 37°C for 15 minutes. The coverslips were then rinsed in PBS, permeabilized for 10 minutes in 0.2% Triton X-100 in PBS, rinsed again in PBS, and blocked with 3% BSA in PBS for 30 minutes. Coverslips to be used for morphological analysis were then stained with a mouse monoclonal anti-βIII tubulin (G712A; Promega, Madison, WI, 1∶2000) followed by an anti-mouse Alexa 488 fluor conjugated secondary antibody (Invitrogen). Coverslips were finally washed again in PBS and mounted in SlowFade Gold antifade reagent with DAPI (Invitrogen, S36938). For cultures stained with actin isoform specific antibodies, coverslips were post-fixed in ice cold 100% Methanol at −20°C for 10 minutes, rinsed for 5 minutes in PBS, and then processed as described above. The directly labeled anti-β-actin antibody described above was used along with a rabbit polyclonal anti-γ-actin 7577 [41] and an anti-rabbit Alexa 568 conjugated secondary antibody. Images were acquired on an Olympus FluoView FV1000 laser scanning confocal microscope or a Zeiss Axiovert 25 micrscope under identical conditions for control and CNS-*Actb*KO coverslips.

Stage 3 neurons were characterized for morphological analysis where the axon was specified as the primary neurite at least twice as long as the next longest primary neurite. All measurements were made with the NeuronJ ImageJ plugin [43]. Data for analysis of morphological parameters in control (*n* = 127 neurons) and CNS-*Actb*KO neurons (*n* = 149) was collected from at least two independent cultures. Axonal branches were defined as processes extending at orthogonal angles to the axon, while distal axon segments were identified as the process that remained parallel to the axonal segment proximal to the branch point as described previously [44].

### Statistics

All data are presented as mean ± standard error of the mean and calculated with GraphPad Prism 5 software (GraphPad Software, Inc.). T-tests were conducted to determine statistical significance when only two groups were compared while one-way ANOVA was used for groups of three or more accompanied by a Tukey post hoc test. A *p*-vale of <0.05 was considered significant.
